# Evolutionary arguments against moral realism: Why the empirical details matter (and which ones do)

**DOI:** 10.1007/s10539-018-9652-0

**Published:** 2018-11-12

**Authors:** Jeroen Hopster

**Affiliations:** 1 Utrecht University, Utrecht, The Netherlands; 2 Janskerkhof 13a, 3512BL Utrecht, The Netherlands

**Keywords:** Metaethics, Evolutionary debunking, Moral realism, Darwinian Dilemma, Reliability challenge, Evolutionary contingency, Historical debunking

## Abstract

The aim of this article is to identify the strongest evolutionary debunking argument (EDA) against moral realism and to assess on which empirical assumptions it relies. In the recent metaethical literature, several authors have de-emphasized the evolutionary component of EDAs against moral realism: presumably, the success or failure of these arguments is largely orthogonal to empirical issues. I argue that this claim is mistaken. First, I point out that Sharon Street’s and Michael Ruse’s EDAs both involve substantive claims about the evolution of our moral judgments. Next, I argue that combining their respective evolutionary claims can help debunkers to make the best empirical case against moral realism. Some realists have argued that the very attempt to explain the contents of our endorsed moral judgments in evolutionary terms is misguided, and have sought to escape EDAs by denying their evolutionary premise. But realists who pursue this reply can still be challenged on empirical grounds: debunkers may argue that the best, scientifically informed historical explanations of our moral endorsements do not involve an appeal to mind-independent truths. I conclude, therefore, that the empirical considerations relevant for the strongest empirically driven argument against moral realism go beyond the strictly evolutionary realm; debunkers are best advised to draw upon other sources of genealogical knowledge as well.

## Introduction

In the recent metaethical debate about evolutionary debunking arguments (EDAs), there has been a tendency to de-emphasize the relevance of genuinely evolutionary considerations for debunkers’ purposes. For instance, Raymond Das describes it as

a deep irony that the import of the specifically *evolutionary* aspect of EDAs is itself fairly minimal. Apart from being (for the most part) a ‘how possibly’ story about the origins of our moral judgments (…), the evolutionary component of such arguments is, as Joyce (2016, p. 125) has noted, ‘strictly, dispensable.’ Any equally plausible causal explanation of our moral judgments that does not presuppose the truth of such judgments would serve the evolutionary debunker’s purposes just as well – or as poorly. (Das 2016, p. 419)

Similarly, referring to the most renowned EDA against moral realism—Sharon Street’s (2006) Darwinian Dilemma—David Enoch submits that

there is nothing essentially Darwinian about the Darwinian Dilemma. Replace any other (non-tracking) causal explanation of why we make the normative judgments that we do in fact make, and the realist will again find herself up against the problem of explaining strong correlations analogous to the ones Street draws attention to. (Enoch 2010, p. 426)

Das, Joyce and Enoch have not been the first ones to question the relevance of the strictly evolutionary aspect of EDAs. In fact, in the concluding section of her article, Street herself makes it explicit that

I have focused on the case of Darwinian influences on our evaluative judgments because I think it raises the problem for realism in a particularly acute form. In principle, however, an analogous dilemma could be constructed using any kind of causal influence on the content of our evaluative judgments. (…) At the end of the day, then, the dilemma at hand is not distinctly Darwinian. (Street 2006, p. 155)

If not as an evolutionary challenge, how should Street’s EDA be understood? Several commentators (Enoch 2010; Clarke-Doane 2012; Crow 2016; Klenk 2017; Tersman 2017; Schechter 2018) have interpreted her argument as the moral analogue of the Benacerraf-Field challenge for mathematical Platonism. Roughly, in its generalized form, this is the challenge of explaining how we can reliably track facts or truths about some specified domain, if we assume that these facts or truths are stance-independent and causally inert. Reframed in the context of moral realism, the challenge is to explain how we have managed to arrive at moral beliefs that are mostly true (hence reliable), assuming—as many moral realists do—that the truth-makers of these beliefs are stance-independent and causally inert facts. Evolutionary considerations are inessential to this challenge; any causal explanation for why we endorse the moral judgments we do suffices to generate it.

I submit that these commentators are right to point out that the Benacerraf-Field challenge is one of the most potent philosophical challenges for moral realism and that part of Street’s work—especially her (2016) practical/theoretical puzzle—relies on a challenge along much of the same lines. But it would be a mistake to identify the Benacerraf-Field challenge with the challenge embodied by the Darwinian Dilemma. One obvious difference between them is that evolutionary considerations are irrelevant to the former, whereas they are crucial to the latter, as I will demonstrate in this article.

These commentators are also right to point out that many EDAs in metaethics, including Street’s Darwinian Dilemma, partly rely on metaphysical and epistemological assumptions. But even if evolutionary considerations—or empirical considerations more generally—don’t do *all* the work in challenges against realism, it would be a mistake to think that they barely do *any* work in them. I will argue that with regard to Street’s Darwinian Dilemma, empirical considerations are central to evaluating its ultimate success. Moreover, I will argue that in order to make the best empirical case against realism, debunkers following Street’s tracks should move beyond the strictly evolutionary realm and invoke historical considerations as well, in order to fashion an adequate response to realists who deny the dilemma’s evolutionary premise (e.g. Shafer-Landau 2012; FitzPatrick 2015; Huemer 2016).

The article proceeds as follows. In Sect. 2 I recapitulate Street’s evolutionary premise and the Darwinian Dilemma it gives rise to. In Sect. 3 I compare and contrast Street’s argument with Michael Ruse’s claim about the evolutionary contingency of our moral attitudes and show how Ruse’s contingency claim gives rise to a slightly different EDA against realism. In Sect. 4 I argue that taken individually, Ruse’s and Street’s respective EDAs each face a specific objection, which they can salvage by drawing upon each other’s arguments. In Sect. 5 I outline a strategy for realists to block these EDAs: they can deny that evolutionary forces have deeply influenced the contents of our endorsed moral judgments. I submit that realists may be right to insist that the contents of some—perhaps many—endorsed judgments cannot be adequately explained in evolutionary terms. However, in Sect. 6 I proceed to argue that this does not safeguard realism from empirically driven debunking arguments: debunkers may still argue that the *best historical explanation* of our moral endorsements involves no appeal to mind-independent moral truths.

## Street’s ‘Darwinian Dilemma’

The target of Street’s Darwinian Dilemma is ‘evaluative realism’, the defining claim of which is that evaluative facts or truths are stance-independent: their truth is not determined by endorsements from any actual or hypothetical perspective.^1^ In what follows I shall discuss a version of her argument with a more constrained scope that is specifically geared towards the *moral* domain.^2^

Street’s dilemma is generated by the empirical hypothesis that evolutionary forces have been a major causal influence shaping our evaluative attitudes (which includes our *moral* attitudes). More specifically, Street (2006, p. 119) claims that our ‘basic evaluative tendencies’ have been shaped by a process of natural selection. These tendencies are our intuitive inclinations—shared with many other species—to perceive certain behaviours as ‘called for’ or ‘demanded by’ the circumstances; for example, a natural urge to protect one’s offspring, or a tendency to seek pleasure and to avoid pain. This is Street’s so-called adaptive-link account of the origins of our evaluative tendencies: these tendencies originated as adaptive responses to environmental circumstances. While the contents of our evaluative tendencies do not strictly determine the contents of our ‘full-fledged evaluative judgments’, Street hypothesizes that they are related nonetheless: ‘[H]ad the general content of our basic evaluative tendencies been very different, then the general content of our full-fledged evaluative judgments would also have been very different, and in loosely corresponding ways’ (Street 2016, p. 120). In a first approximation, then, Street’s empirical premise is that natural selection has had a substantial -– albeit indirect—influence on our endorsed evaluative judgments.

Why would this empirical premise, if true, be troublesome for moral realists? Realists typically assume that the set of moral judgments that people endorse largely coincides with the set of stance-independent moral truths. But Street argues that this assumption cannot be maintained in the light of the evolutionary influences that permeate the contents of our moral judgments. This is what the first horn of her Darwinian Dilemma is meant to establish: assuming that there are stance-independent moral truths, we probably fail to track them.

Why so? Street confronts realists with the dilemma of specifying whether there is a relation between stance-independent moral truths and the evolutionary influences on our moral judgments:

- either the realist holds that there is *no* relation between the evolutionary forces that have influenced the contents of our moral judgments and the independent moral facts or truths
- or the realist holds that there *is* such a relation.

Both horns of the dilemma leave realists in an unappealing position, Street argues. On the first horn of the dilemma, realists must either explain how the contents of our moral judgments happened to coincide with the contents of the stance-independent truths or regard the evolutionary influences on these judgments as ‘distorting’—i.e. grant that these influences have resulted in judgments that are probably *false*, assuming realism. Simply *positing* that they coincide is question-begging, Street maintains: the realist needs to *explain* this coincidence. This ‘demand for an explanation’ is better understood as a demand for theoretical justification: realists need to show how *it follows from their theory* that our evolutionarily influenced moral judgments probably coincide with the stance-independent moral truths. But if realists deny that there is a relation between the evolutionary forces that influence our judgments and these independent truths, then it seems very difficult to provide such justification.

Realists who accept the second horn of the dilemma and grant that there *is* a relation between the evolutionary forces that have shaped our judgments and mind-independent moral truths are committed to a truth-tracking explanation: presumably, the correlation between our moral judgments and these independent moral truths is best explained by the hypothesis that it tended to be reproductively advantageous for our ancestors to make true moral judgments. But Street argues that this truth-tracking explanation is scientifically inferior to her adaptive-link account, according to which moral truths are ultimately a construction of our evolved attitudes. Our moral judgments don’t tend to be fitness-enhancing because we have tracked mind-independent truths that correlate with reproductive success. Instead, we tend to regard fitness-enhancing judgments as true, because this tendency increased the reproductive success of our ancestors. Hence, as an inference to the best explanation, moral realism is untenable.

### The reliability challenge

The first horn of the Darwinian Dilemma can be reframed as a ‘reliability challenge’ for moral realism that is meant to establish the following conclusion: assuming realism, we have no guarantee that our moral judgments are generally reliable. To elucidate this challenge, we may frame it in terms of sets. Realists assume that the contents of the moral judgments that we endorse roughly coincide with the contents of stance-independent moral truths. But Street argues that given the vast number of alternative judgments that we *could* have endorsed, such a coincidence would be very implausible. Consider the various moral judgments that we do not endorse—from the judgment that ‘infanticide is laudable’ to the judgment that ‘plants are more valuable than human beings’ to the judgment that ‘the fact that something is purple is a reason to scream at it’ (Street 2006, p. 133). If the realist denies that there is a relation between the influence of natural selection on our judgments and the independent moral truths, then given the vast range of logical possibilities, it would be very remarkable if natural selection accidentally shaped our judgments in concordance with these truths.^3^ In all likelihood it has not, Street concludes.

How might realists respond to this challenge? Some have done so by challenging the relevance of the possibilities that Street invokes. Since Street is presenting an *internal* challenge to realism—assuming realism, we have no guarantee that our judgments are reliable—the judgments relevant to her challenge are the judgments we could make *assuming realism*. But not all realists grant that all logical possibilities are relevant, assuming realism (e.g. Berker 2014, p. 246; FitzPatrick 2014, p. 253; Wielenberg 2010, pp. 458–459). If the relevant possibility space can be strongly diminished, for example by showing that the possibilities Street envisions are unintelligible (FitzPatrick 2014) or conceptually deficient (cf. Cuneo and Shafer-Landau 2014), then Street’s claim that our judgments are likely to be off-track loses much of its force. Perhaps the rough alignment of our moral judgments with the independent moral truths will still be *somewhat* of a coincidence, but not nearly as unlikely as Street takes it to be.

The *desideratum* for realists who take on the first horn of Street’s dilemma, then, is to argue that a coincidence is sufficiently likely to justify the assumption that our moral judgments are generally reliable. Realists can do so by arguing that the set of possible moral judgments we *could* make, assuming realism, is much smaller than Street suggests. However, it won’t help the realist to merely *posit* that the set of possible judgments we could endorse is limited and clearly correlated with the set of judgments that are evolutionarily beneficial. Street’s explanatory demand is to justify *why* this is the case—i.e. to offer a theory-driven consideration, assuming realism, for limiting the relevant possibility space. To offer such a consideration *after* having granted that there is no relation between the evolutionary influences on our moral endorsements and the contents of mind-independent moral truths seems very difficult. In effect, by granting that evolutionary influences do not constrain the possibility space of candidate mind-independent moral truths, realists *do* commit themselves to the thesis that these truths might have been *anything*. The more promising option for realists, then, is to argue that there *is* a relation between the mind-independent moral truths and our evolutionarily shaped judgments. But this brings us back to the second horn of Street’s dilemma: realists can only frame this as a truth-tracking relation, which should be discarded on scientific grounds, or so Street argues.

## Ruse’s contingency challenge

In Ruse’s (1995) work we find two metaethical challenges for moral realism. In this section I briefly discuss both of them and zoom in on the latter challenge, which relies on an assumption about the evolutionary contingency of moral norms.

According to Ruse, morality can be regarded as a biological mechanism for fostering cooperation. Our moral sense is an adaptation, which is all the more effective in fulfilling its evolutionary function because it creates the illusion that morality derives from objective foundations external to ourselves. This is what makes morality work: we obey moral norms precisely because we take them to be objective. But the suggestion that morality has a stance-independent foundation is in fact illusory, as its evolutionary origins make clear.

How does Ruse’s evolutionary hypothesis challenge realism? We can distinguish between two strands in Ruse’s work. Sometimes, Ruse invokes the metaethical premise that given the evolutionary explanation of our moral beliefs the objective foundation of morality has to be judged redundant:

You would believe what you do about right and wrong, irrespective of whether or not a ‘true’ right and wrong existed! The Darwinian claims that his/her theory gives an entire analysis of our moral sentiments. Nothing more is needed. Given two worlds, identical except that one has an objective morality and the other does not, the humans therein would think and act in exactly the same ways. (1995, p. 254)

In this passage, what does the work in Ruse’s EDA against realism is the assumption that a Darwinian explanation of our beliefs in objective moral truths makes the existence of their truth-makers metaphysically redundant: we no longer need to posit them. If our best evolutionary explanation nowhere supposes that our moral beliefs track objective truths, Ruse suggests, then we should erase these objective truths from our ontology.

Elsewhere Ruse advances a different evolutionary argument against realism. If we assume that ‘objective morality’ corresponds to some reality external to human beings, Ruse argues, then

we might have evolved in such a way as to miss completely its real essence. We might have developed so that we think we should hate our neighbors, when really we should love them. Worse than this even, perhaps we really should be hating our neighbors, even though we think we should love them! (Ruse 1995, p. 242)

Here Ruse relies on the idea that our value-judgments are evolutionary contingent: we might have evolved making very different value-judgments. Given this possibility, how do we know that our actual value-judgments are correct? If the range of possibilities implied by the contingency of our values is sufficiently large, then we should conclude that the reliability of our actual endorsements cannot be guaranteed; they are probably false. Hence, Ruse’s argument can be reframed as a reliability challenge for realism much like that of Street, but framed in terms of the contingency of our moral judgments.

Some recent commentators have framed Ruse’s challenge somewhat differently: as targeting the *epistemic safety* of our moral beliefs (e.g. Bogardus 2016, p. 645). Even if we assume that our current moral beliefs reflect the contents of stance-independent truths, the *ease* with which we might have evolved different moral beliefs—beliefs that would be false, assuming realism—seems troubling for realists.^4^ This ease suggests that our moral beliefs lack the property of being epistemically ‘safe’, assuming realism:

**Safety:** S’s beliefs about a domain D are safe iff there is no nearby world in which S, using similar cognitive means to those in our actual world, arrives at false beliefs about D.

Safety is generally (though not universally) accepted as a necessary condition for knowledge.^5^ The underlying idea is that if S’s beliefs about a domain are unsafe—they could easily have been false—then their truth is merely a matter of luck. This realization seems to undercut the justification of S’s beliefs about D. Or in the context of moral realism: it provides an undercutting defeater for the hypothesis that our moral beliefs coincide with mind-independent moral truths. Justification may be reinstated, but the burden of proof is with realists to show that it can be.

Whether Ruse’s EDA is framed as a challenge for the truth of our moral judgments or the safety of our moral beliefs, assuming realism, on both versions the success of his argument hinges on the claim that our moral endorsements are evolutionarily contingent, which is -– at least in part—an empirical claim. More precisely, for his EDA to succeed, our moral endorsements should be contingent in a way that is troublesome for realism: the contingency of our endorsements should imply that either they are probably false, or that they might have easily been false, assuming realism.

## Entangled between Street’s and Ruse’s EDAs

In some respects, Ruse’s evolutionary challenge for realism is quite similar to Street’s Darwinian Dilemma. Both EDAs challenge, on evolutionary grounds, the assumption that our moral judgments track mind-independent truths, and both of them do so, at least in part, by presenting a reliability challenge for moral realism. But the challenges differ in the details. What motivates Ruse to question the reliability of our moral judgments, assuming realism, is that realism allows for the possibility that we might have evolved in such a way as to completely miss the mind-independent moral truths. This claim presupposes that our moral judgments are evolutionarily contingent; if they are, then either our moral judgments are false or they might easily have been, assuming realism.

By contrast, for the success of Street’s argument it is irrelevant whether or not our evolved moral beliefs are contingent in an *evolutionary* sense. Even if they are not—i.e. even if from an evolutionary point of view, our moral beliefs could not have been any different—Street can still raise her challenge for moral realism: to account for the striking correlation (striking, that is, in the light of the vast space of what is logically possible) between the moral judgments that we take to be true and judgments that tend to contribute to our reproductive success. Hence, what motivates Street to question the reliability of our moral judgments, assuming realism, is that realists do not have a proper explanation, informed by their own theory, for the assumed coincidence between our moral judgments and the mind-independent moral truths. What seems to be the only possible realist proposal to explain this coincidence—to advance a truth-tracking account—is untenable on scientific grounds.

While Street’s Darwinian Dilemma does not depend on a claim about evolutionary contingency, in this section I will demonstrate that such a claim might nonetheless help her argument. I will argue that some of the most popular responses that realists have offered to Street’s dilemma—so-called third-factor accounts—lead to contingency worries like those that Ruse invokes. Moreover, I will argue that one potent reply against Ruse’s EDA—an appeal to evolutionary constraints—arguably reinforces the empirical hypothesis underlying Street’s Darwinian Dilemma. Hence, by advancing Street’s and Ruse’s respective EDAs in tandem, debunkers can deflect some common realist replies.

### Countering third-factor objections

First, consider some prominent third-factor accounts that realists have advanced in reply to Street’s dilemma. Third-factor theorists submit that there is a relation between the evolutionary influences on our moral judgments and the independent moral truths, but argue that this relation is indirect: there is some third-factor involved which indirectly causes our basic evaluations and grounds their truth. In order to come up with a third-factor explanation, realists have to make some modest assumptions about which evaluations are in fact true. For instance, David Enoch (2010, p. 430) bases his third-factor explanation on the assumption that ‘survival is at least somewhat good’. Since evolution ‘aims’ at survival, what evolution aims at is at least somewhat good. Another third-factor account, proposed by Knut Olav Skarsaune (2011, p. 230), starts from the assumption that ‘pleasure is usually good and pain is usually bad’. Since natural selection has generally led us to seek pleasure and to avoid pain, it has caused us to value things that tend to be good.

The alleged upshot of third-factor accounts is to explain why it need not be regarded as a striking coincidence that our evolved moral endorsements correlate with the independent moral truths. But it is questionable whether the accounts of Enoch and Skarsaune succeed in showing this. One of the difficulties that their accounts face is that the modest moral assumptions they invoke allow for the evolution of various kinds of moral judgments, many of which realists will presumably regard as false.^6^ For instance, there is a wide variety of behaviours that can be survival-enhancing, including cheating, stealing, free-riding and making self-serving moral judgments.^7^ Likewise, there is a variety of behaviours that can induce pleasure, including making jokes at the expense of helpless others or eating factory-farmed animals. Presumably, realists will regard the moral judgments that accompany such behaviours, or at least many of them, as off-track: they do not track independent truths. However, it is not the third-factor explanation that tells us so; indeed, these are all behaviours that the proposed third-factor explanation might allow for. As a result, third-factor theorists once again face a reliability challenge: of all the moral judgments we could have made in the light of the proposed third-factor explanation, what guarantees that our actual judgments coincide with the mind-independent truths?

This challenge is structurally similar to the first horn of Street’s Darwinian Dilemma. The debunker argues that realists need to show that *it follows from their theory* that our evolutionarily influenced moral judgments probably coincide with the stance-independent moral truths, and that third-factor theorists like Enoch and Skarsaune have failed to show this. But in addition to Street’s original EDA, the reliability challenge now explicitly relies on considerations about evolutionary contingency. If the realm of moral judgments that we might evolve, assuming realism, is merely constrained by the assumption that survival is somewhat good, or that pleasure is usually good and pain usually bad, then the reliability of our actual judgments cannot be guaranteed. The realist has established a relation between the evolutionary influences on our judgments and the independent truths, but she has not sufficiently constrained the relevant possibility space: replay the tape of life and we might evolve survival-enhancing judgments with very different contents. Enoch’s and Skarsaune’s accounts still allow for the possibility that we might have evolved to love our neighbours, whereas, really, we should hate them.

### Countering objections regarding evolutionary constraints

Now turn to Ruse’s contingency challenge. One way in which realists have challenged Ruse’s EDA is by questioning the evolutionary contingency thesis on which it relies. Ruse illustrates the supposed contingency of our moral judgments by invoking counterfactuals that involve species with phenotypes very different from our own. For instance, he asks us to imagine that

instead of evolving from savannah-living primates (which we did), we had come from cave dwellers. (…) Or take the termites (to go to an extreme example from a human perspective). They have to eat other’s feces, because they lose certain parasites, vital for digestion, when they molt. Had humans come along a similar trail, our highest ethical imperatives would have been very strange indeed. (Ruse 1995, pp. 241–242)

This counterfactual is reminiscent of a passage by Darwin, who speculated in *The Descent of Man* that

if men were reared under precisely the same conditions as hive-bees, there can hardly be a doubt that our unmarried females would, like the worker-bees, think it a sacred duty to kill their brothers, and mothers would strive to kill their fertile daughters; and no one would think of interfering. (Darwin 2013, p. 58)

Such evolutionary counterfactuals may be suggestive, but how plausible are they? Realists might counter that a capacity to make moral judgments is only evolvable for species quite similar to ourselves—e.g. species with a language-infused capacity for making judgments and deliberating about different courses of action, a cognitive capacity for norm-guided behaviour and an emotional sensitivity for the well-being of others. Hence, rather than accepting that our moral judgments are evolutionarily contingent, realists might argue that there are developmental or adaptive constraints which limit the possible contents of such judgments. If this counterargument holds up, then Ruse’s contingency argument does not get off the ground: if we could not have easily evolved as a species with a moral capacity while endorsing very different moral norms, then we could not have easily missed morality’s ‘real essence’. As a result, we would have no reason to question the truth or justification of our moral beliefs, assuming realism.

The biologist Jeffrey Schloss (2014) has recently argued along these lines. Schloss holds that there are strict structural and developmental constraints on the evolution of a moral capacity, which limit the resultant contents of judgments that a species with a moral capacity might feasibly endorse. These constraints include not only high levels of sociality and intelligence, but also ‘life expectancy, mortality rate, fertility rate, body size, and the relationship between degree of infant dependence, parental care, lifelong pair bonding, group hunting, and even bipedal gate (which modified the pelvis resulting in increased dependence, care, and pair bonds)’, according to Schloss (2014, p. 110). This combination of features does not evolve easily—and neither does a capacity for moral judgment. The fact that on our planet this capacity has evolved only in one species suggests as much.^8^ If Schloss is correct, then we should expect that the evolution of a moral sense in any species will be closely tied to capacities that are distinctive of human beings. Counterfactuals like those of Ruse, Schloss submits, may be ‘as impossible for biology as a square circle is for geometry’ (idem, p. 109).

Schloss’s argument, if correct, helps realists to block Ruse’s contingency challenge. But it also serves to reinforce the general empirical claim underlying Street’s Darwinian Dilemma—namely, that evolutionary forces have been a major causal influence shaping our moral attitudes. While Street (2006) herself gives substance to this evolutionary claim by advancing an adaptationist explanation of the contents of our moral judgments, her appeal to natural selection is not strictly necessary for the purposes of her EDA: debunkers following Street’s approach merely need to provide evidence that evolutionary forces—however specified—have played a major causal role in shaping our judgments. Since Schloss’s appeal to developmental constraints serves to corroborate this empirical hypothesis, it leads to the Darwinian Dilemma: realists have to explain how the evolutionary forces that have influenced our moral judgments are related with the stance-independent moral truths.

In sum, by borrowing elements from both Street’s and Ruse’s respective EDAs, debunkers can enhance their best argument against moral realism. Street’s dilemma can be extended by an appeal to evolutionary contingency, which serves to refute some of the most popular third-factor accounts. An evolutionary rebuttal of Ruse’s contingency thesis, in turn, serves to corroborate the evolutionary premise of the Darwinian Dilemma.

## Denying influence: A solution for realists?

Might realists be able to resist the debunker’s argument? Perhaps the most promising strategy for realists is to take a more radical stance and to resist the premise that the contents of our moral judgments have been saturated with evolutionary influence. In this section I present the best case for realists who pursue this strategy. I will argue that some realists are indeed well positioned to resist Street’s and Ruse’s empirical claims and to argue that evolution’s causal influence in shaping our moral endorsements has been relatively minor. As I will argue in the next section, however, realists who pursue this strategy are still committed to a controversial empirical claim—namely, that over the course of human history we have been able to track mind-independent moral truths.

### The truth-tracking hypothesis

Consider how realists who deny the empirical premise of the Darwinian Dilemma might think of the relation between evolution and our moral judgments. These realists may grant that evolutionary forces have had a moderate influence on our moral endorsements, in two respects. First, they may submit that evolutionary influences have thoroughly influenced the contents of *some* of our moral judgments, but submit that this influence does not generalize. Second, they may grant Street’s claim that natural selection has shaped the contents of our *basic* moral evaluations, but resist the further claim that this influence also affects our *full*-*fledged* moral endorsements. Instead, an adequate explanation of the latter should involve an additional factor: the process of grasping stance-independent moral truths.

Indeed, as it stands there may be good grounds for questioning Street’s adaptive-link hypothesis: the hypothesis our moral judgments can be explained in evolutionary terms, since our basic evaluative tendencies—e.g. feelings of pleasure and pain, and the accompanying intuitions about what is to-be-pursued and what is to-be-avoided—originated as adaptive responses to environmental circumstances. Even if our basic moral tendencies evolved as adaptive responses, it remains to be shown that they also provide the main source of input for our *full*-*fledged* judgments—and it is questionable whether they do in fact provide this input. Street acknowledges that our full-fledged judgments can ‘stray, perhaps quite far, from alignment with our more basic evaluative tendencies’ (Street 2006, p. 120), and stresses that it ‘is likely that we were selected above all else to be extremely flexible when it comes to our evaluative judgments’ (Street 2006, p. 158, fn. 20). But if our full-fledged moral judgments are rather different from our basic proto-moral tendencies, then Street’s adaptive-link account fails to explain, for the most part, the contents of our full-fledged moral endorsements.

Several realists (e.g. Shafer-Landau 2012; FitzPatrick 2015; Huemer 2016) have pursued a reply to EDAs along these lines, arguing that the contents of many archetypical moral judgments cannot obviously be explained in evolutionary terms. Consider moral judgments that foster inclusionary values, for example the judgment that all human beings ought to be treated respectfully, irrespective of their capacities or group membership, or the judgment that non-human animals should be treated as subjects of moral consideration (cf. Buchanan and Powell 2015). These value-judgments have only won widespread adherence in recent human history, which raises some doubt over the suggestion that their contents have been deeply influenced by evolutionary forces. Moreover, it is not obvious that such judgments are fitness-enhancing.

The most important factor shaping our moral endorsements, these realists maintain, is our capacity to grasp stance-independent truths. How did this capacity originate? Some realists have sought to explain it as a by-product of our general emotional and intellectual capacities. These capacities, perhaps stimulated by the emergence of human language and our subsequent capacity to systematically reflect upon and reason about our moral judgments, have enabled us to track truths of a mind-independent reality. FitzPatrick (2015, p. 889), for example, regards this truth-tracking capacity as an ‘intelligent extension of evolutionarily influenced evaluative judgment’.

If this account is along the right lines, then realists are well positioned to criticize EDAs on empirical grounds. First, consider Ruse’s contingency EDA. If rational moral reflection, fuelled by the recognition of stance-independent moral truths, has been the dominant factor shaping our moral judgments, then the contents of these judgments are likely to be invariant with respect to our species-typical characteristics. As a result, Ruse’s suggestion that our value-judgments are evolutionarily contingent loses much of its plausibility. Whatever biological inclinations Darwin’s hive-bees or Ruse’s termites might have, this need not translate into what they regard as a ‘sacred duty’ or ‘ethical imperative’. If hive-bees would indeed make moral judgments very different from ours, then their rational faculty has probably been ill-calibrated: their judgments are mistaken.

Next, consider Street’s Darwinian Dilemma. If we have indeed evolved a capacity to track stance-independent moral truths, then the first horn of Street’s dilemma is unproblematic for realists: the general reliability of our moral judgments is trivially guaranteed. Moreover, if the influence of natural selection, as well as other evolutionary forces, on the contents of our moral judgments turns out to be modest, then on the second horn of the dilemma, Street’s evolutionary explanation is no longer obviously superior to the realist’s truth-tracking account. After all, there is no longer a strong correlation between our evolved judgments and moral truths, and the weaker this correlation, the easier it will be for realists to explain it.

### Divide-and-conquer strategy

As previously stated, realists who maintain that the best explanation of our moral endorsements is that we have grasped stance-independent moral truths may still grant that evolution has influenced the contents of *some* of our moral judgments. Whether this influence has been distorting or not—i.e. whether evolutionary forces have pushed our judgments towards or away from the independent moral truths—should be decided on a case-by-case basis. Accordingly, realists may advance a divide-and-conquer strategy against debunkers (cf. Berker 2014) by partitioning our moral endorsements into different subsets and independently considering their justificatory status:

- Some moral judgments (e.g. ‘We ought to foster the well-being of future generations’) have hardly been shaped by evolutionary forces. Instead, they are primarily products of our capacity to reflect on stance-independent moral truths. This is particularly plausible for moral judgments in favour of inclusionary moral commitments, which are typically beyond the scope of evolutionary explanations (Buchanan and Powell 2015).
- Some moral judgments have been shaped by natural selection but simultaneously reflect stance-independent moral truths. The earlier-mentioned judgment that ‘pleasure is generally good and pain generally bad’ might be such a judgment. Arguably, something similar is true for several moral judgments that are explicable in terms of kin selection (‘If forced to choose, you ought to favour the well-being of your own children over the well-being of strangers’), reciprocal altruism (‘The fact that someone has treated you well is a reason to treat that person well in return’) or indirect reciprocity (‘The fact that someone is an example to society is a reason to admire her’). Note that for this part of the divide-and-conquer strategy to work, realists still have to dismantle the second horn of Street’s dilemma and argue for the superiority of a tracking hypothesis over an adaptive-link account (see Artiga 2015 and Deem 2016 for such attempts).
- A third class consists of moral judgments that are the product of evolutionary forces which have led us away from the moral truths. For instance, ethicists typically argue that judgments fuelled by racial bias or by moralized disgust reactions are morally off-track. Evolutionary psychologists typically argue that such responses are largely explicable in evolutionary terms. Hence, realists may argue that these judgments belong to a class of evolved responses that should be regarded as generally unreliable.

## Best historical explanation: A comeback for debunkers?

Realists who submit that the act of grasping mind-independent truths might play a legitimate—and important—role in causal explanations of our moral endorsements, and who are not impressed by the evidence that purports to show that these endorsements have been influenced by evolutionary forces, are well positioned to put pressure on EDAs. But as I will argue in this section, debunkers might be able counter this argument by shifting their empirical considerations from the evolutionary to the historical domain. Even if our full-fledged judgments have not been saturated with evolutionary influences, they may still be saturated with other causal influences—causal influences that typically belong to the explanatory domain of historians. If the best historical explanation of our moral endorsements nowhere involves an appeal to the fact that over the course of human history we have tracked mind-independent moral truths, then the realist’s truth-tracking hypothesis should be abandoned on historical grounds, or so debunkers might argue.

### Historical debunking argument

How does this historical debunking argument relate to Street’s EDA? As noted in the previous section, Street (2006) herself favours an adaptive-link account of the origins of our moral judgments. But she also seems to be aware of the limitations of this account and is sympathetic to the view that our basic attitudes are only weak determinants of our full-fledged judgments. Indeed, she highlights that apart from the indirect influence of natural selection, our full-fledged judgments have also been shaped by various other processes, such as social learning and deliberation, as well as the *sui generis* influence of rational reflection (see Street 2006, esp. sects. 4 and 5).

If these various non-evolutionary factors play a major role in shaping the contents of our moral judgments, then it is not obvious that the Darwinian Dilemma can get off the ground. But the ‘best explanation challenge’ embodied in the second horn of the dilemma need not be articulated in evolutionary terms. Debunkers can appeal to our best scientific explanations more broadly—particularly to explanations from the field of history—to argue that non-realist theories are superior on empirical grounds. The crucial condition for such explanations to have any debunking force is that they show that the best, scientifically informed genealogies of our moral endorsements involve no appeal to stance-independent moral truths.

Importantly, this debunking argument will only be problematic for realists who maintain that mind-independent moral truths have causal powers and that appealing to these truths is an integral part of the best causal explanation of our moral endorsements. But this, we have seen, is indeed the view of realists who deny the empirical premise of the Darwinian Dilemma (Shafer-Landau 2012; FitzPatrick 2015; Huemer 2016). These realists are committed to a *scientific hypothesis*—namely that we have been able to track, over the course of human evolution or history, stance-independent moral truths. In other words, by positing the existence of stance-independent moral truths and arguing that these truths cause our judgments, these realists enter the game of scientific explanation. Assuming that compatibility with our best scientific explanations is regarded as an important metaethical *desideratum*, if it turns out that this hypothesis cannot be corroborated—or is even clearly invalidated—by our best science, this will count as highly damaging to these realist positions.

### Genealogies without stance-independent truths

Do the best, scientifically informed genealogies of our moral endorsements indeed involve no appeal to stance-independent moral truths? Answering this question in appropriate detail requires a historical case study of specific moral endorsements, which is beyond the scope of the present discussion. Still, to give an impression of how debunkers might proceed, consider the following brief sketch of a genealogy of inclusionary moral values.

In *The Descent of Man*, Darwin observed that the process of civilization comes along with a broadening of human’s moral concern:

As man advances in civilization, and small tribes are united into larger communities, the simplest reason would tell each individual that he ought to extend his social instincts and sympathies to all the members of the same nation, though personally unknown to him. This point being once reached, there is only an artificial barrier to prevent his sympathies extending to the men of all nations and races. (Darwin 2013, p. 76)

Indeed, many contemporary moral judgments promote inclusionary values. How did this extension of moral concern come about, if not by grasping the stance-independent truth that ‘men of all nations’ deserve equal respect?

Debunkers may argue as follows. First, even if inclusionary moral judgments have not directly been shaped by natural selection, they may still be *indirectly* saturated with evolutionary influence. For instance, there may be an evolved empathy response underlying our inclusionary judgments which has been co-opted in the process of moral reflection and has subsequently been extended beyond kith and kin. Second, debunkers are not committed to the claim that the *only* input in this process of moral reflection comes from our evolved attitudes. The input is likely to be much broader and to include our socioculturally developed attitudes and factual knowledge, as well as the norm of impartiality that typically guides moral reflection—a norm whose origins may plausibly be explained in evolutionary terms (cf. DeScioli and Kurzban 2013). Third, debunkers may point out that it took a notoriously long time before sympathies were extended to ‘men of all nations and races’, and that even nowadays the boundaries of our moral concern are contested. This observation should not surprise us if moral judgments are largely the products of our evolved inclinations, lessons learned from history and ongoing social dialogue. By contrast, the observation seems difficult to reconcile with the hypothesis that we have grasped the stance-independent moral truth that all people deserve equal respect.^9^

### Making up the balance

A crucial difference between the accounts of realists and debunkers rests on the question of whether the best explanation of our moral beliefs involves an appeal to stance-independent truths. This brings us back to the question with which we began—namely, which empirical considerations are relevant to the success of EDAs. If my argument has been along the right lines, then this empirical input is not limited to evolutionary considerations. In fact, it may well turn out that the most revealing test for the empirical tenability of moral realism is to scrutinize the *historical* genealogy of our moral endorsements in detail and to evaluate whether the relevant data are best explained with or without appealing to stance-independent moral truths. To provide such a detailed genealogy, and to evaluate its fit with a realist versus a non-realist metaethics, will be an important task for future metaethical inquiry.

## Conclusion

I have argued that the evolutionary considerations that Street and Ruse invoke can reinforce each other in presenting the evolutionary debunker’s strongest case against moral realism. For realists who try to block Street’s EDA by proposing a third-factor account based on a general moral assumption, the contingency of the moral judgments that are evolvable in the light of this assumption seems problematic, as it allows debunkers to reinstate a version of the reliability challenge. For realists who try to block Ruse’s EDA by arguing that the contents of our moral judgments are evolutionarily convergent or constrained by developmental factors, the strong causal influence of evolutionary forces on our moral judgments thereby implied seems problematic, as it reinforces the general evolutionary claim underlying Street’s Darwinian Dilemma.

Perhaps the most promising reply of realists to evolutionary debunkers is to deny that evolutionary forces have saturated the contents of our full-fledged moral judgments. Several realists have insisted that these contents are better explained by our recognition of stance-independent moral facts. In order to support this thesis, realists have to vindicate a scientific hypothesis: over the course of evolution and human history, we have been able to track stance-independent moral truths. Since the thesis that we have been able to track stance-independent truths is key to a success epistemology of moral realism, vindicating this hypothesis is of crucial importance to its overall metaethical standing. Debunkers, in turn, may argue that the best historical explanation of our moral endorsements involves no appeal to this tracking thesis. What counts as the best historical explanation of the contents of our moral endorsements, then, will be crucial for settling this debate.

## Footnotes

1 Street’s most obvious target among realist views is evaluative non-naturalism—roughly, the view that evaluative facts or truths are not reducible to natural facts and do not figure in causal explanations. Street (2006, section 7) also claims to target some naturalist views, but grants that other naturalists escape her challenge. On her taxonomy, the latter views do not count as genuinely realist, since they do not subscribe to the view that moral facts or truths are stance-independent. The term ‘stance-independence’ comes from Shafer-Landau (2003, p. 15), who characterizes it as follows: ‘The moral standards that fix the moral facts are not made true by virtue of their ratification from within any given actual or hypothetical perspective.’ In this article I use the terms mind-independence and stance-independence interchangeably.

2 I submit that the Darwinian Dilemma, understood as an argument that relies on truly evolutionary considerations, becomes more interesting when it is restricted to the moral domain. This is because much of the evidence in support of the hypothesis that many of our moral judgments can (at least indirectly) be evolutionarily explained is not—or not obviously—available with regard to many of our non-moral evaluative judgments.

3 In later writings Street speaks of *conceptual* possibilities (Street 2008) or conjures all *conceivable* possibilities (Street 2016). *Mutatis mutandis*, the same considerations apply to them.

4 The notion of ‘ease’—and the corresponding ‘proximity’ of possible worlds—is difficult to make precise. A potential strategy for doing so in an evolutionary context is to advance a variation on Stephen Jay Gould’s thought experiment *replaying the tape of life*: ‘nearby worlds’ are worlds that might ‘easily evolve’ if we could replay life’s tape.

5 Bogardus (2016, p. 647) himself criticizes the safety condition.

6 Debunkers have criticized third-factor accounts on other grounds as well. Here I restrict my discussion to the question of whether they can restore the reliability of our moral beliefs. Note that my criticism applies specifically to third-factor accounts which rely on highly general moral assumptions.

7 Braddock (2016) lists several examples of such behaviours, which have evolved over the course of human evolution. Presumably, realists maintain that the judgments fuelled by these ‘nasty norms’ are—and have always been—false.

8 That morality has evolved only in one species does not imply that it has evolved only once. In fact, a moral capacity may well have evolved in several human populations.

9 Realists may, of course, reply that it is very difficult to discover moral facts, and they may seek to explain why some of these facts have only been discovered recently (cf. Huemer 2016). The question, then, becomes whether this rival explanation is compatible with our best historical knowledge and superior to the debunker’s account.
